# Overwintering aggregations are part of *Hippodamia undecimnotata*’s (Coleoptera: Coccinellidae) mating system

**DOI:** 10.1371/journal.pone.0197108

**Published:** 2018-06-13

**Authors:** Eline Catherine Susset, Jean-Louis Hemptinne, Etienne Danchin, Alexandra Magro

**Affiliations:** 1 Université Toulouse 3 Paul Sabatier, CNRS, ENSFEA, UMR5174 EDB (Laboratoire Évolution et Diversité Biologique), Toulouse, France; 2 CNRS, Université Paul Sabatier,UMR5174 EDB, Toulouse, France; 3 ENSFEA, Université Toulouse 3 Paul Sabatier, CNRS, UMR5174 EDB (Laboratoire Évolution et Diversité Biologique), Castanet-Tolosan, France; USDA Agricultural Research Service, UNITED STATES

## Abstract

Aggregation during diapause is a common phenomenon in arthropods that nevertheless remains poorly understood. The most commonly claimed benefit is that survival is higher in aggregations but animal aggregations could also be driven by sexual selection. In this perspective, we investigated whether aggregations in insects could be part of their mating system. We studied the overwintering aggregations of the ladybird *Hippodamia undecimnotata* (Schneider), an aphidophagous species from Southern and Eastern Europe as well as Asia. We collected ladybirds at three aggregation sites in Southwest France, during two overwintering periods (2013–2014 and 2014–2015). We checked their reproductive status by counting the viable sperm cells in the sperm storage organs of both males and females, and by assessing the ovarian status of females. We also investigated if mating behaviour occurred in these aggregations. We found that males have a high quantity of viable sperm cells (70–95%) in their reproductive organs throughout the overwintering periods. In contrast, although most females (85–95%) had empty spermatheca at the onset of the aggregations in autumn, the majority (65–91%) had numerous viable sperm in their spermatheca at the time of dispersal from the aggregation in early spring. Furthermore, frequent copulations were observed towards the end of the overwintering period, few weeks before dispersal. These results suggest that finding sexual mates may have been involved in overwintering aggregations in *H*. *undecimnotata*.

## Introduction

Group living is a common feature in the animal kingdom [1–3] and animal aggregations are often temporary and seasonal. In arthropods, seasonal aggregations generally consist of monospecific clusters, sometimes of high numbers of individuals for several months, and are located in the same places year after year [e.g. 4–9]. One of the most famous cases is the spectacular meetings of the monarch butterfly *Danaus plexippus* (L.) (Lepidoptera: Nymphalidae) in the forests of Mexico and Florida [5]. Nevertheless, although seasonal aggregations are a fascinating phenomenon, ultimate mechanisms for their formation are still poorly understood [10–12].

Seasonal aggregations are often concomitant with diapause (a hormonally-mediated arrest of development at a species-specific ontogenic state [13]), which is considered an insect’s main strategy to escape unfavourable seasons [14,15]. A first general assumption is that gathering in the course of diapause increases the protection of the relatively immobile individuals against predators [4,16] owing, for instance, to increased intensity of aposematic signals [17,18] or dilution effects [19]. Aggregations during diapause may also generate favourable microclimates [20,21]: they were shown to promote water conservation [22–24] or increase temperature inside the group [23]. However, although the most common evolutionary approach underlying the formation of aggregations in insects has focused on survival, there are other major determinants of fitness that have been overlooked [4,25,26].

The hidden lek hypothesis states that animal aggregations can be driven by sexual selection and result from the settlement of promiscuous males on sites where they are most likely to encounter female mates [27–29]. According to that hypothesis, by joining one particular site males and females are not looking for aggregation *per se* but for high quality individuals of the opposite sex [4]. This was shown to be the case for some animals [30–32]. This view does not exclude that aggregations then convey other benefits but it posits that these benefits are not at the very origin of aggregations [33]. Although this hypothesis was developed for vertebrates, it is also valid in insects as female mating preferences are sometimes responsible of the formation of clusters in flies and other insects [34].

Worldwide, many ladybird (Coleoptera: Coccinellidae) species aggregate in response to seasonality (see reviews in [9, 35]), either hibernating and/or aestivating. Conventional wisdom has it that ladybirds aggregate either to regulate their microclimate, or to accentuate the aposematic signal sent to the predators, thus increasing survival (e.g. [33]). Nevertheless, no study has tested whether aggregated ladybirds survive better. Interestingly, Taylor (1984) [36] and Majerus (1994) [33] also mentioned that ladybirds could mate during overwintering aggregations. With Hagen (1962) [35] and Hodek and Landa (1971) [37], they noted that copulations often occur in overwintering aggregations in several species. Moreover, some authors found that females in aggregations had no sperm in the spermatheca at the beginning of diapause and that the proportion of females with sperm gradually increased over the course of the overwintering period [37–40]. In addition, Hodek and Landa (1971) [37] showed that males had no or few spermatocytes in testicular follicles but had their seminal vesicle full of sperm cells from the beginning to mid-overwintering period. Then, males had fully mature spermatocytes in both their testicular follicles and their seminal vesicle at the end of the aggregation period. Their results are supported by Hemptinne and Naisse’ observations (1987) and Ceryngier et al. (2004) [39,40] who found that most males had inactive or intermediately active testes during the course of overwintering period; Hemptinne and Naisse (1987) [39] also noted that seminal vesicles were always full. That is, although there are observations that point out to the existence of mating during overwintering aggregations, there is no throughout study of its importance.

Our hypothesis is that ladybird overwintering aggregations can be part of their mating system, in the perspective of the hidden lek hypothesis. Indeed, being in the aggregation before dispersing in spring towards breeding sites should greatly increase odds of finding and choosing a compatible sexual partner. Here, as a first step to test that hypothesis, we investigated the extent to which and when mating occurs at the aggregation sites. To do so, we chose the ladybird *Hippodamia (Semiadalia) undecimnotata* (Schneider) as a model. This species has several synonyms (*Ceratomegilla undecimnotata*, *Semiadalia undecimnotata*) currently being used by different authors. Ślipiński (2007) [41] mentions there is indeed some lack of consensus about the generic limits in the *Hippodamia*-generic complex. Here we follow Fürsch (2007) and Iablokoff-Khnzorian (1982) [42,43] taxonomies as we did in previous papers on the same topic [44,45].

*H*. *undecimnotata* is an aphidophagous ladybird species from Southern and Eastern Europe, and Asia [43]. It exhibits large overwintering aggregations at the base of prominent objects located at the top of promontories [9,36,45,46]. Individuals gather at specific and long-used locations year after year eventually undertaking long migrations from breeding and feeding sites in lowlands to aggregation sites [9,36]. Depending on the climate and/or geographical range, overwintering of *H*. *undecimnotata* in its whole range takes place between September and May and lasts 5 to 9 months [9,36]. Shortening of day length, reduction of food availability and quality, and a general drop in temperatures induce diapause [47–49].

We performed the first comprehensive, systematic study of both the proportion and viability (a precise measure of reproductive status not used before) of sperm cells in males and females, ovarian status of females and mating behaviour of *H*. *undecimnotata*, in several clusters, at different aggregation sites during two overwintering periods.

## Material and methods

### Study sites

The study took place over two consecutive overwintering periods at three sites in Southwest France: Labastide-Gabausse (LG, latitude: 44°2’N, longitude: 2°6’E, altitude: 260 m a.s.l.), Saint-Michel-de-Lanès (SML, latitude: 43°19’N, longitude: 1°45’E, altitude: 315 m a.s.l.), and Mont Seigne (MS, latitude: 44°12’N, longitude: 2°55’E, altitude: 1128 m a.s.l.). The study was carried out on private lands with the permission of the owners.

LG is an organic vineyard and ladybirds aggregate in cracks on the vine stocks present at the highest points. SML is situated on a conventional crop area dominated by wheat and maize and ladybirds aggregate at the base of a phone pole next to a road. MS is a heathland dominated by *Calluna vulgaris* Hull (Ericaceae), with low human disturbance during the winter and ladybirds aggregate in the crevices of two orientation tables.

In the study sites and in both overwintering periods, *H*. *undecimnotata* arrived at the aggregation sites from late October to early-November depending of the sites, and left from mid-April to late April. So, the study took place from November 2013 to April 2014 (Year 1), and from November 2014 to April 2015 (Year 2).

### Field sampling of ladybird

We sampled ladybirds in the 3 aggregation sites, from the beginning to the end of the overwintering periods. Samples were taken in November, January, March, and April in both Years 1 and 2. For each aggregation site, samples were collected in two clusters at least 10 m apart from each other, except at SML where only 1 cluster was available.

We carefully collected the ladybirds with fine tweezers to avoid disturbing the remaining ladybirds. After sampling, we brought them to the laboratory in a portable fridge at 10°C and they were individually stored in vials in a climate-controlled room (10°C, Light:Darkness ratio 0:24 for 24h) until dissections.

Two hundred and ninety-seven males and 340 females and 408 males and 438 females were analysed in Year 1 and in Year 2, respectively.

### Dissection of ladybirds and sperm collection

We sexed ladybirds under a stereo-microscope according to the shape of the distal abdominal sternites. We carried out dissections in a cavity slide the day following the sampling. The ventral tegument was teased apart with fine tweezers, and 100 μl of a phosphate-buffered saline (PBS) were added to avoid cytolysis and organ retraction. In males, we removed the reproductive organs (i.e. the testes and the seminal vesicle); in females, we removed the spermatheca and the *bursa copulatrix*. The remains of the ladybird body were removed from the cavity slide. We ruptured the whole reproductive organs to allow sperm release. As the spermatheca is a chitinous structure, we pressed it with forceps to eject the sperm. Then, we pipetted 100 μl of PBS containing the sperm from the cavity slide into a microcentrifuge tube. We washed the cavity slide with PBS (400 μl for males; 100 μl for females) that was added into the microcentrifuge tube. The tube was then vortexed at 600 RPM for 30 min. This allowed remaining sperm cells to leave the reproductive organs without increasing sperm mortality [50].

### Sperm cell number

We counted sperm cells under a dark field phase contrast microscope at 400x magnification. We counted the number of sperm cells in two samples for each individual; count repeatability was high (Pearson’s correlation test: r = 0.82; P < 0.0001). The number of sperm cells detected in both samples was thus summed and multiplied by the dilution factor to estimate the total number of sperm cells. Twenty to 25 males and females were analyzed per sampling period for each site.

### Sperm cell viability

**Fig 1:**
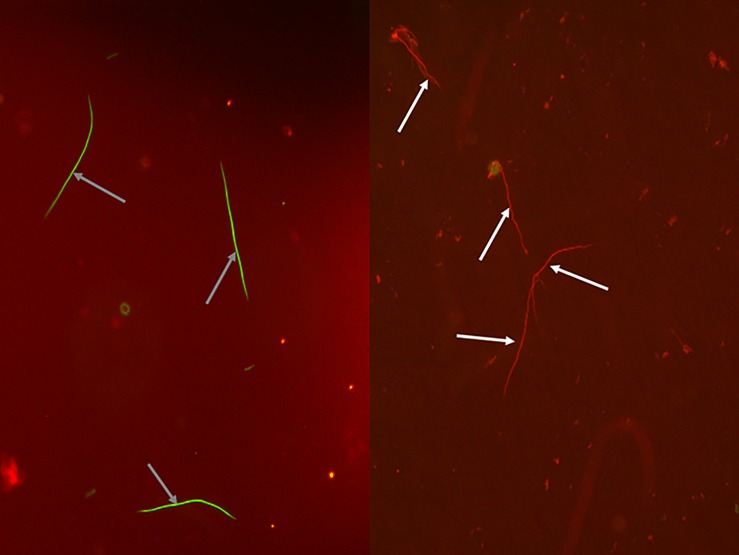
Assay of sperm cell viability. Live sperm cells are stained in green (indicated by grey arrows), dead sperm cells are stained in red (indicated by white arrows).

Sperm cell viability assays were carried out following Damiens et al. (2002) [51] and García-González and Simmons (2005) [52]. We used the LIVE/DEAD^®^ Sperm viability reagents (Molecular Probes). Ten μl of sperm were pipetted and mixed with an equal volume of 1:50 diluted 1 mM SYBR-14 (a permanent nucleic stain), and were left in the dark for 10 min before 2 μl of 2.4 mM propidium iodide was added. The sample was then incubated in the dark for 10 min and observed under a fluorescence microscope (blue excitation filter at λ = 490 nm). Live sperm cells were green stained with SYBR-14, a membrane permanent nucleic acid stain, while dead ones (i.e. with damaged membranes) were stained red with propidium iodide [51–52] (Fig 1). To assess the repeatability of the measurement, sperm cells viability (the proportion of viable to non-viable sperm cells) was assessed in two samples for each of 21 ladybirds, and was found to be high (Pearson’s correlation test: r = 0.79; P < 0.0001).

### Ovarian status

We investigated the ovarian status for each female for which we had sperm cell numbers and viability. Ovarian development was scored on a 1 to 5 scale according to Okuda and Hodek (1989) [53]: stage 1: inactive ovary with ovarioles containing no oocytes; stage 2: ovarioles with the transparent oocytes; stage 3: ovarioles with small, vitellogenic oocytes; stage 4: ovarioles with vitellogenic oocytes; stage 5: ovarioles with mature oocytes.

### Exhaustive sampling of females at the time of dispersal at LG site

We investigated the proportion of females leaving the aggregation site in a mated condition in a cluster that had not been used for the other parts of the study, at the LG site. We caught all dispersing ladybirds by means of an interception entomological trap. This trap, inspired by Sarthou (2009) [54], is an asymmetric tent with no ground sheet. The trap was set up to enclose a vine and was fitted tightly to the ground. It was set up in March 2014 and 2015 when the ladybirds were still immobile and densely packed, and removed in May once all the ladybirds had dispersed. The emerging insects were collected in a bottle at the top of the trap. The collecting bottle was checked 3 times a week and emptied when at least one ladybird was trapped. Ladybirds were brought back to the laboratory and their spermatheca and *bursa copulatrix* content analysed the following day, as described above. The results of this sampling correspond to the data given for LG in April in the “Number and viability of sperm cells in females” part of the Results section.

### Ladybird behaviour at the LG aggregation site

We observed ladybird behaviour at each sampling period and once a week on a sunny day, from March to the beginning of May 2014 and 2015, at Labastide-Gabausse between 9:00 am and 6:00 pm. We performed control visits once a month during raining days to check whether mating occurred only during sunny days. In 2014, we followed two clusters of about 200 and 70 ladybirds each and recorded movements across the aggregation and mating events. In 2015, we followed one cluster of about 155 ladybirds. Mating ladybirds were marked with white paint (Edding^®^) on the elytra; paint dots lasted at least 2 months on elytra.

### Statistical analyses

We analysed the differences in the proportion of males and females having viable sperm cells in their reproductive organs, as well as the differences in sperm cell number and viability, and the ovary maturation according to the Site, Period, Year, and Cluster variables with Generalized Linear Mixed Models (GLMMs). We assumed a binomial error distribution for the proportion of individuals with sperm cells and for sperm viability [55]. We used a penalized quasi-likelihood GLMM (glmmPQL) with quasi-Poisson error distribution for the sperm cell number; we investigated the differences in female ovary maturation with a GLMM assuming a Poisson error distribution. In all models, Period was entered as fixed effect while Site, Year, and Cluster were considered as random factors. Period was a 4-level ordered variable: November, January, March and April. Due to heavy rainfalls in March 2014, the aggregation site at SML was destroyed. So, the Site variable had only 2 levels in Year 1: LG and MS and 3 levels in Year 2: LG, MS, and SML.

In addition, we investigated the differences in the sperm cell number and viability in the females sampled in the last sampling period (i.e. April) according to Site, Year, and their interaction using a Generalized Linear Model (GLM) with a quasi-Poisson and a binomial error distribution, respectively to compare the number and viability of sperm cells across sites and years, respectively.

All analyses were conducted in the R (version [2.14]) environment [56], using the lme4 [57] and MASS [58] packages.

## Results

### Numbers and viability of sperm cells in males

The proportion of males with sperm cells in their reproductive organs was high: between 78% and 100% of males had sperm cells in their reproductive organs (Table 1). Most of the time, all males had sperm cells in their reproductive organs (Table 1). The proportion of males with sperm cells in their reproductive organs did not vary in relation to the period of sampling (GLMM: χ^2^_3_ = 4.50, P > 0.05).

**Table 1:** Sperm cells in male reproductive organs (sperm cell number/male). Proportion of males with sperm cells, sperm cell number and viability during the two successive overwintering periods at the 3 study sites.

Sites	Period	N	Males with sperm cells (%)	Sperm cell number (mean ± SE)	Sperm cell viability (mean ± SE, %)
*Year 1 (2013–2014)*					
LG	Nov.	45	86.7	165,867 ± 91,111	73.23 ± 3.06
	Jan.	44	91	459,116 ± 64,733	84.51 ± 2.72
	March	39	100	523,788 ± 55,685	80.48 ± 4.03
MS	Nov.	41	78	131,183 ± 70,873	89.30 ± 2.56
	Jan.	42	100	67,700 ± 6,189	84.26 ± 1.88
	March	41	97.5	570,386 ± 64,061	85.52 ± 2.41
	April	45	97.7	489,912 ± 51,284	72.48 ± 3.78
*Year 2 (2014–2015)*					
LG	Nov.	41	100	431,141 ± 35,176	88.2 ± 2.35
	Jan.	49	100	390,397 ± 38,797	88.2 ± 1.11
	March	48	100	277,929 ± 25,201	92.5 ± 1.09
MS	Nov.	41	100	421,745 ± 35,445	85.8 ± 3.10
	Jan.	51	98	299,648 ± 40,137	90.5 ± 1.60
	March	40	100	227,702 ± 27,766	90.5 ± 1.85
	April	49	91.8	280,671 ± 27,638	91.8 ± 1.50
SML	Nov.	20	100	416,705 ± 53,609	90.6 ± 2.49
	Jan.	23	100	490,003 ± 35,349	77.4 ± 2.16
	March	25	100	665,913 ± 473,465	91.4 ± 1.22
	April	21	85.7	222,505 ± 28,728	91.9 ± 2.65

N = Number of sampled males.

For Year 1, sperm cell number was the highest during the second half of the overwintering period. In Year 2, it was high in November 2014 and declined progressively until March and April 2015. However, the number of sperm cells increased in March 2015 before declining again in April at SML (Table 1). Nevertheless, when analysing only the correlation between the Period and the number of sperm cells in males, we found that the number of sperm cells did not vary according to the Period (quasi-Poisson GLMM: χ^2^_2_ = 2.04, P > 0.05).

Finally, the viability of sperm cells in males ranged between 72.48 ± 3.78% and 92.5 ± 1.09% (Table 1). The viability of sperm cells in males did not vary between the periods of sampling (GLMM: χ^2^_3_ = 0.33, P > 0.05).

### Number and viability of sperm cells in females

The proportion of females with sperm in the spermatheca and the *bursa copulatrix* varied between the sampling periods (GLMM: χ^2^_3_ = 12.02, P < 0.01). The proportion of females having sperm cells in their spermatheca and *bursa copulatrix* increased over the winter. The general trend was clear: few females were mated at their arrival at the aggregation sites. This persisted until March, and then most females were mated in April (Table 2). The exhaustive collection of departing females at LG showed that 91% and 83% out of 124 and 47 females in April 2014 and April 2015 respectively had sperm in their spermatheca and the *bursa copulatrix*.

**Table 2:** Sperm cells in female spermatheca and *bursa copulatrix* (sperm cell number/female). Sperm cell number and viability during the two successive overwintering periods at the 3 study sites.

Sites	Period	N	Mated females (%)	Sperm cell number (mean ± SE) per mated female	Sperm cells viability (mean ± SE, %)
*Year 1(2013–2014)*					
LG	Nov.	43	9.3	4765 ± 2808	71.2 ± 15.9
	Jan.	42	4.7	6100± 1910	68.48 ± 15.48
	March	42	40.5	15186 ± 2747	91.73 ± 2.15
	April	124	91.1	8988± 1222	86.85 ± 2.58
MS	Nov.	35	2.9	100	100
	Jan.	42	2.4	2270	85.28
	March	45	15.6	14185± 4161	74.24 ± 12.96
	April	41	65.9	5720± 542	84.04 ± 3.12
*Year 2(2014–2015)*					
LG	Nov.	45	15.6	6714 ± 3380	88.1 ± 4.17
	Jan.	52	19.2	3200± 753	91.7 ± 5.14
	March	51	23.5	5440± 2400	93.6 ± 2.71
	April	47	83.0	3650± 711	93.7 ± 1.6
MS	Nov.	45	6.7	3517 ± 1343	93.3 ± 6.7
	Jan.	42	4.8	9250 ± 8750	92.5 ± 7.5
	March	50	8.0	6900 ± 2866	91 ± 3.87
	April	46	80.4	6222 ± 832	92.9 ± 1.15
SML	Nov.	20	5.0	3750	90.6
	Jan.	24	12.5	2417 ± 843	89.02 ± 5.48
	March	20	10.0	3500 ± 2500	90 ± 10
	April	25	76.0	7277 ± 1541	92.5 ± 2.01

N = Number of sampled females.

Regarding the total sperm cell numbers in the spermatheca and the *bursa copulatrix* in April we found a significant Site × Year interaction (quasi-Poisson GLM: F_1,142_ = 8.56; P < 0.01). In Year 1, LG females had a higher sperm load than MS females whereas in Year 2, LG females had the lowest sperm load compared to MS and SML females. The viability of sperm cells in April was also related to the Site × Year interaction (GLM: F_1,139_ = 26.08; P < 0.001). Nevertheless, sperm cell viability in the spermatheca and *bursa copulatrix* was high (> 80%) at the time of dispersal in both years (Table 2).

### Ovarian status

**Fig 2:**
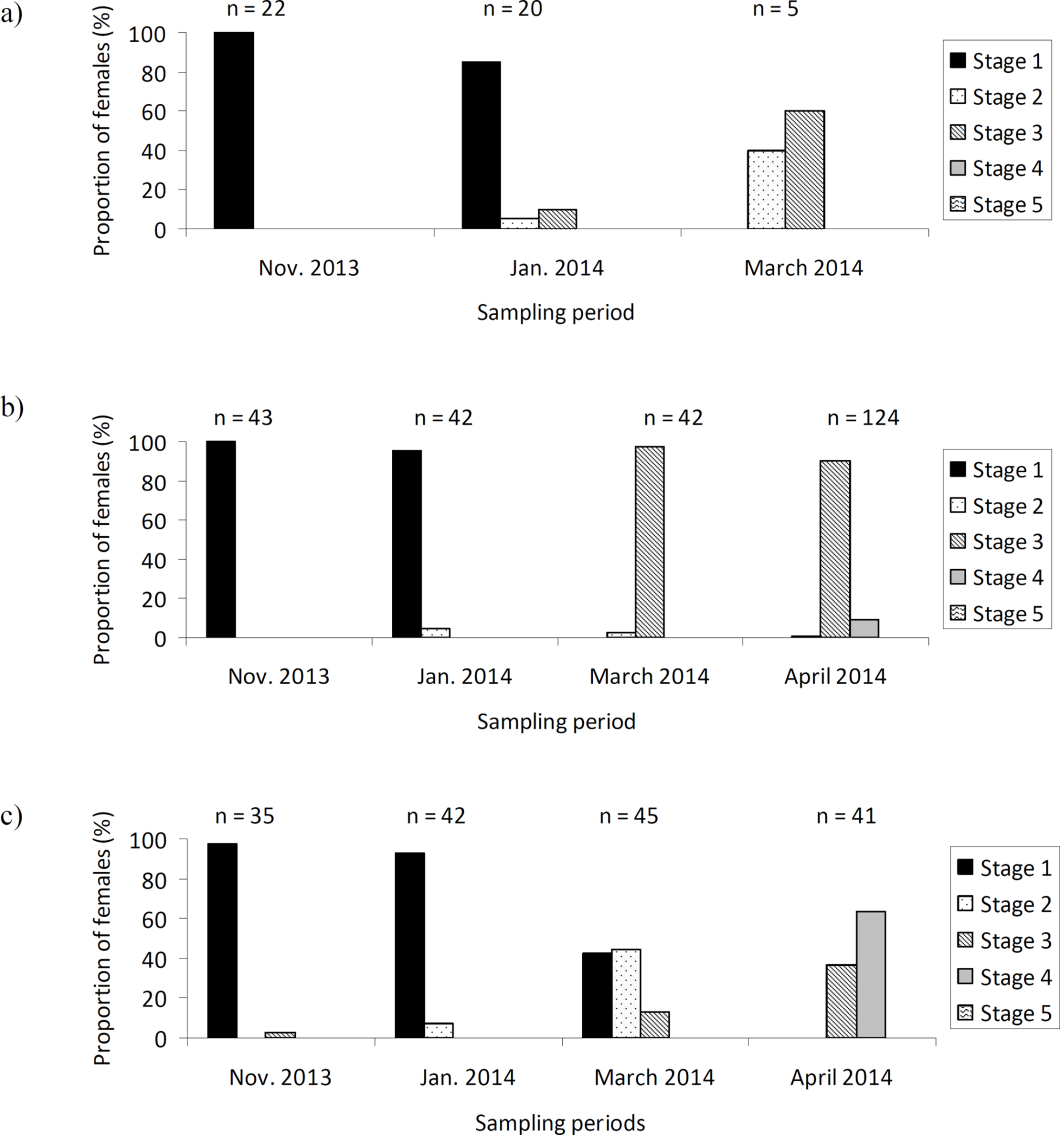
**Stages of maturation of the ovaries of *Hippodamia undecimnotata* in relation to the sampling locations and periods 2013–2014** at a) SML, b) LG, and c) MS. In March 2014, only 5 females were sampled at SML due to site destruction.

**Fig 3:**
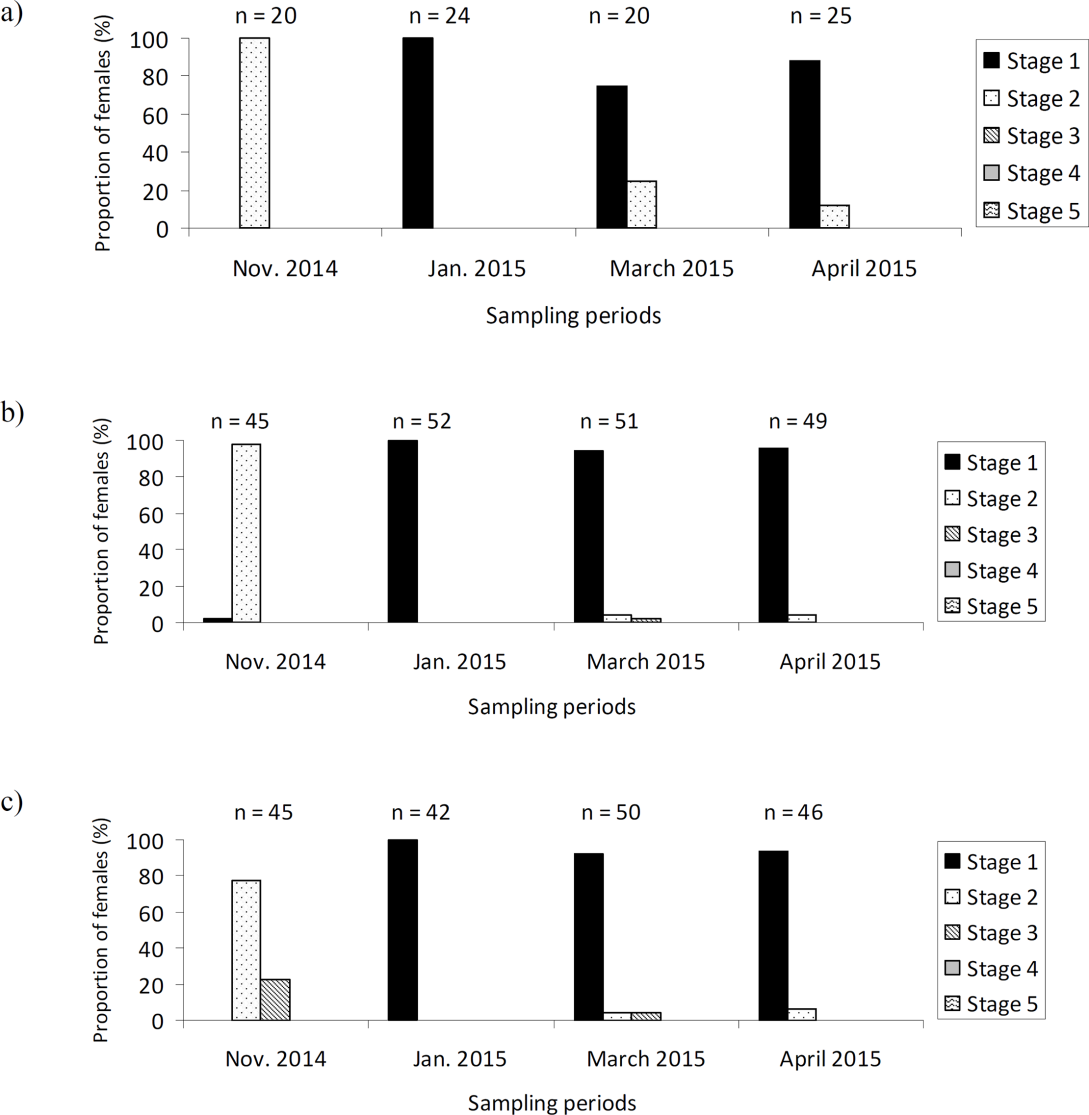
**Stages of maturation of the ovaries of *Hippodamia undecimnotata* in relation to the sampling locations and periods 2014–2015** at a) SML, b) LG, and c) MS.

The ovarian status varied between the sampling periods (GLMM: χ^2^_3_ = 28.46; P < 0.001). In Year 1, females had immature ovaries (below stage 3) from their arrival at the aggregation site until January 2014 in all 3 locations. A higher proportion of females had ovaries containing vitellogenic oocytes in March. All dissected females in April 2014 had ovaries with vitellogenic oocytes (stage 3 and beyond) (Fig 2). In Year 2, although some females had ovaries with vitellogenic oocytes (i.e. at stage 3) in November, the majority of them had immature ovaries all over winter and spring even when leaving the aggregation (Fig 3).

### Ladybird behaviour at the LG aggregation site

In both 2014 and 2015, the first walking ladybirds were observed at the beginning of February. Before that, ladybirds remained immobile and densely packed inside the crevices. The first copulations took place in March.

In 2014, over 16 non-consecutive days (representing a total of 144 hours of observation), a total of 46 and 21 different couples out of more than 219 and 208 individuals were recorded in cluster 1 and cluster 2, respectively. No mating was observed during raining days (over 40 hours of observation).

In 2015, over 14 non-consecutive days (representing 126 hours of observations), a total of 25 couples out of about 155 individuals were recorded.

## Discussion

The evolutionary significance of the overwintering aggregations of arthropods is far from clear. We tested the hypothesis that arthropods usually scattered in breeding and feeding habitats aggregate during the harsh season to mate before dispersing towards breeding sites. In this perspective, we thoroughly studied the sexual activity of *H*. *undecimnotata* during the overwintering aggregation.

### Most males at the aggregation sites had sperm cells

We found that 78 to 100% of the males had several hundreds of thousands of viable sperm cells in their reproductive organs for the whole of the two consecutive overwintering periods. Interestingly, we found that males from all the clusters arrived at the aggregation sites with viable sperm cells, indicating they had mature reproductive organs and were thus physiologically able to inseminate females. These results are in accordance with observations from Hodek and Landa (1971) [37] and Ceryngier et al. (2004) [40] for the same species: they found testis resorption but seminal vesicle filling during the first part of the aggregation period. The *H*. *undecimnotata* populations studied by those authors come from Central Europe and these differences might be due to ecological plasticity in terms of dormancy as it happens for *C*. *septempunctata* [59].

Finally, we noticed that in most cases sperm cell numbers decreased just before dispersal in year 2 which may suggest that many mating events occurred at the aggregation. Sperm production is energetically costly [60], and therefore we hypothesize that if males mated they might have had no opportunity to renew their sperm stock at the aggregation site.

### Females arrived with empty tracts but left loaded with sperm cells

At the onset of the overwintering period in November, most females had an empty spermatheca and *bursa copulatrix* indicating they were not mated. For the less than 15% having sperm cells in these organs, we suggest two explanations. First, those females had been inseminated during the rare early copulations observed in the beginning of the aggregation. Alternatively, one cannot exclude that some of those females might have been mated during the previous breeding season or even during the previous overwintering period, several months before, ladybirds being able to store sperm for up to 8 months [61]. Indeed, some *H*. *undecimnotata* might survive long enough to join the aggregation sites more than once as it was reported for other ladybird species such as *Harmonia axyridis* (Pallas) or *Henosepilachna pustulosa* (Kôno) (see [62–64] and review in [9]).

We also found that the proportion of females with sperm cells increased over the winter, reaching 65 to 91%, upon leaving the aggregations. These results confirm previous observations made in one population by Hodek and Landa (1971) [37] and Ceryngier et al. (2004) [40]. Moreover, our original study of sperm quantity and viability in females’ reproductive tracts showed an increase in the number of sperm cells throughout the overwintering period. Therefore, our results strongly suggest that mating takes place before leaving the aggregation.

### Mating was common at the aggregation sites toward the end of the overwintering period

We observed that mating occurred at the aggregation sites, in agreement with the observations of Hodek and Landa (1971) [37], Tanaka et al. (1987) [65], and Ceryngier et al. (2004) [40] incidental observations. It happened despite the fact that most females had immature ovaries. El Hariri (1966), Hodek and Landa (1971), Hemptinne and Naisse (1987) and Ceryngier et al. (2004) [37,39,40,66] had previously observed that ovaries stay immature during the overwintering period. These results are in accordance with the fact that ovarian maturation is not a prerequisite for the onset of mating in coleopterans [65,67].

More importantly, the frequency of mating in March-April just before aggregation breaks was particularly high. It happened when both sperm cell number and viability in male gonads and female organs were the highest. These results further suggest that mating is a significant activity at the aggregation sites before dispersal to breeding sites.

Altogether, our results strongly support the hypothesis that overwintering aggregations are part of the mating strategy of *H*. *undecimnotata*.

### Aggregation site features and mating system characteristics

By aggregating and mating during the overwintering period on the top of promontories [9,36,45], *H*. *undecimnotata* behaves like hilltopping insects [67]. Hilltopping is a mate-locating strategy akin to a lek [67,68] in which insects head for topographical summits or prominent features to meet with their potential mates [67]. The constancy of the aggregation sites and their prominent features, comparable to landmarks, probably ease mate finding [69]. This mating strategy should be more thoroughly investigated for *H*. *undecimnotata* and should be more thoroughly investigated. For instance, it remains to be studied if the sex-ratio in ladybirds is biased at the beginning of the overwintering period, which is a key feature of the hilltopping system [70].

### Conclusion: are overwintering aggregations sexually driven?

It is frequently explained that arthropods aggregate in winter or the dry season because they would survive better. The adaptive nature of this behaviour was the paradigm for the evolution of animal aggregations, and remained accepted uncritically. However, the observation that mate choice occurs in and has the potential to drive bird colonies and lizard groups opens other perspectives [71,72]. More generally, Danchin and Wagner (1997) [25] suggested that the origin of animal aggregations may be a by-product of selection acting on the many choice processes necessary to survive and breed rather than on the direct advantages of aggregation. That ladybird’s sexual activity takes place so intensely at the aggregation sites in winter lends some support to Danchin and Wagner’s views, and is a strong incentive to further explore the mating system of these insects in relation with aggregation behaviour.
